# Perceived Discrimination and Self-Rated Health in Europe: Evidence from the European Social Survey (2010)

**DOI:** 10.1371/journal.pone.0074252

**Published:** 2013-09-05

**Authors:** Javier Alvarez-Galvez, Luis Salvador-Carulla

**Affiliations:** 1 Department of Social Policy, Faculty of Social Sciences and Law, Universidad Loyola, Andalucía, Seville, Spain; 2 Centre for Disability Research and Policy, Faculty of Health Sciences, University of Sydney, Sydney, Australia; Public Health Agency of Barcelona, Spain

## Abstract

**Introduction:**

Studies have shown that perceived discrimination has an impact on our physical and mental health. A relevant part of literature has highlighted the influence of discrimination based on race or ethnicity on mental and physical health outcomes. However, the influence of other types of discrimination on health has been understudied. This study is aimed to explore how different types of discrimination are related to our subjective state of health, and so to compare the intensity of these relationships in the European context.

**Methods:**

We have performed a multilevel ordered analysis on the fifth wave of the European Social Survey (ESS 2010). This dataset has 52,458 units at individual level that are grouped in 26 European countries. In this study, the dependent variable is self-rated health (SRH) that is analyzed in relationship to ten explanatory variables of perceived discrimination: color or race, nationality, religion, language, ethnic group, age, gender, sexuality, disability and others.

**Results:**

The model identifies statistically significant differences in the effect that diverse types of perceived discrimination can generate on the self-rated health of Europeans. Specifically, this study identifies three well-defined types of perceived discrimination that can be related to poor health outcomes: (1) age discrimination; (2) disability discrimination; and (3) sexuality discrimination. In this sense, the effect on self-rated health of perceived discrimination related to aging and disabilities seems to be more relevant than other types of discrimination in the European context with a longer tradition in literature (e.g. ethnic and/or race-based).

**Conclusion:**

The present study shows that the relationship between perceived discrimination and health inequities in Europe are not random, but systematically distributed depending on factors such as age, sexuality and disabilities. Therefore the future orientation of EU social policies should aim to reduce the impact of these social determinants on health equity.

## Introduction

Perceived discrimination has an impact on our physical and mental health [1–5]. Evidence shows that people perceiving themselves as subject to discrimination can suffer mental problems such as depression, psychological distress, anxiety, phobias or high-risk health behaviors [6]. In fact, there are even cases where people suffering from these psychological problems resort to suicide as a way to escape from this social problem [7–9]. However, individually perceived discrimination has also been associated with specific physical health problems including hypertension, breast cancer, self-reported poor health, and other potential risk factors such as obesity, high blood pressure or substance abuse that can deteriorate our health [10].

A relevant part of literature has highlighted the influence played by race or ethnicity discrimination on mental and physical health outcomes [4–11]. Race is based on physical differences (e.g. skin color, facial features, etc.), while ethnicity is a broader concept that emphasizes differences in language, cultural traditions, learning behaviors or customs. Despite the conceptual differences of race and ethnicity are social determinants of health that, in practice, are generally associated with stressful experiences among ethnic minorities. Conflicting cultural relationships related to immigration and experiences of discrimination based on race and ethnicity are often positively related to adverse (mental) health status [12]. For instance, studies have shown that immigrant population, which frequently is represented by discriminated ethnic minority groups, is associated with greater health inequalities. Despite social changes in modern advanced societies, these groups still face significant barriers that complicate their full social integration with native populations and, ultimately, influence their poor-health indicators [13]. However, in daily life there are many other types of perceived discrimination that can determine an individual’s state of health. Factors such as gender, sexual orientation, language, religiosity, nationality, social class, or disabilities can also lead to poor health outcomes [11].

Discrimination, as an action based on prejudices, may be defined as the differential treatment of individuals based on arbitrary or ascribed characteristics that are socially attributed to belonging to that group, including traits as diverse as gender, sexual orientation, age, education, intellectual or physical disabilities, belief and religion, race and ethnicity, political orientation, or even socio-economic background [14]. However, the literature has not paid the same attention to all these social determinants of health. Perceived discrimination based on race and ethnicity has a long tradition in literature [15–22]; nevertheless recent studies have found that non-racial discrimination based on gender, education or age are also relevant predictors of health outcomes [11,23]. In this line, Kim and Williams demonstrate a consistent association between different perceived discriminatory experiences and poor self-rated health in South Korea [11]. The findings of this study in South Korea suggest that specific types of discrimination based on different social statuses –such as gender, age, education level, disability, birth region, and so on – are significantly associated with poor self-rated health. In this case, education level and age were found to be the main sources of perceived discriminatory experiences.

At the beginning of the XXI century, European societies have had to face different challenges, among many others: the control of irregular immigration flows, the ageing process of the European population, the extension of equal opportunities to people with physical/intellectual disabilities, the chronic unemployment of modern societies and the deterioration of labor markets in the current context of the financial crisis. People with disabilities today represent over 15% of the EU population and they still represent a discriminated collective that does not have equal opportunities in modern societies [24]. The number of old people is growing rapidly in developed societies as is age discrimination in accessing the labor market [25,26]. Furthermore, immigrant workers are subject to increased discrimination during economic downturns like today’s [27]. Thus the question addressed in this work is the following: how are these different types of perceived discrimination related to health inequalities in European countries?

According to this general query, the present study aims to explore the association between experience of discrimination and self-rated health in European countries. Specifically, the purpose of this work is to examine how different types of discrimination are related to our subjective state of health, and so to compare the intensity of these relationships in the European context. Our specific objectives are the following: (a) to describe the prevalence of discriminatory experiences in Europe; (b) to identify the associations between these experiences and self-rated health in European countries; (c) to compare the intensity of these social determinants in order to explain differences in individuals’ self-rated health.

In relation to these objectives, our hypotheses are the following:

• H1: The prevalence of perceived discrimination in Europe varies depending on socio-demographic and socio-economic determinants such as gender, age, marital status, education, household income or domicile size.
• H2: Different forms of perceived discrimination are related to different health outcomes in European countries. That is, the effect of different types of perceived discrimination over self-rated health of people living in Europe depends on the context.
• H3: The effect of perceived discrimination on self-rated health related to aging and disabilities can be more relevant than other types of discrimination in the European context.

## Materials and Methods

### 2.1: Data and Variables

In order to explain the prevalence of discriminatory experiences in Europe and their association with health outcomes, we have analyzed the fifth wave of the European Social Survey (ESS 2010) [28].

This dataset has a sample size of 52,458 units at individual level. At contextual level, the 26 countries included in the fifth wave of the ESS 2010 are the following: Belgium, Bulgaria, Cyprus, Czech Republic, Denmark, Estonia, Finland, France, Germany, Greece, Hungary, Ireland, Israel, Lithuania, Netherlands, Norway, Poland, Portugal, Russian Federation, Slovakia, Slovenia, Spain, Sweden, Switzerland, Ukraine and the United Kingdom. Turkey and Romania were not included in this study since in ESS-5 there is no available data for these countries. The target population of this survey covered individuals over 15 years of age who are residents within private households, regardless of nationality or citizenship, language or legal status. A complete description of the ESS 2010 is provided elsewhere (visit the following link: http://ess.nsd.uib.no/ess/round5/).

In this study, the dependent variable is self-rated general state of health (SRH) which was included in the ESS 2010 questionnaire in question C15 (“How is your health in general? Would you say it is...”), with a 5-point Lickert scale where: 1 “Very bad”, 2 “Bad”, 3 “Fair”, 4 “Good”, and 5 “Very good”.

Ten explanatory variables of perceived discrimination were included in the analysis (see Table 1). Specifically, these variables were perceived discrimination by color or race, nationality, religion, language, ethnic group, age, gender, sexuality, disability and other. These binary variables correspond to question C25 in the questionnaire (“On what grounds is your group discriminated against?” where 1 means “discrimination of respondent’s group” in the different response categories). In addition to these variables referring to different types of perceived discrimination, another six related to socio-demographic and socio-economic aspects were included as controls: gender (where ‘male’ was the reference category), age (encoded in six intervals: 0 ‘15-24’, 1 ‘25-34’, 2 ‘35-44’, 3 ‘45-54’, 4 ‘55-64’, 5 ‘65 or more’), marital status (where 0 ‘married or civil union’, 1 ‘divorced’, 2 ‘widowed’, 3 ‘single’), domicile (where 0 ‘rural village’, 1 ‘small city’, 2 ‘suburbs in big city’ or 3 ‘big city’), education (where 0 ‘primary’, 1 ‘lower secondary’, 2 ‘upper secondary’, 3 ‘tertiary’) and household income (where 0 ‘quartile 1’, 1 ‘quartile 2’, 2 ‘quartile 3’, 3 ‘quartile 4’). These variables were included in the model because both socio-demographic and socio-economic determinants have been found relevant in explaining health inequalities.

**Table 1:** Descriptive statistics for variables in the model.

**Variable**	**Obs.**	**Mean**	**Std. Dev.**	**Min.**	**Max.**
Self-rated health	52,379	3.724	0.968	1	5
Discr. Color or race	52,458	0.010	0.102	0	1
Discr. Nationality	52,458	0.014	0.117	0	1
Discr. Religion	52,458	0.011	0.103	0	1
Discr. Language	52,458	0.007	0.081	0	1
Discr. Ethnic group	52,458	0.010	0.101	0	1
Discr. Age	52,458	0.009	0.096	0	1
Discr. Gender	52,458	0.005	0.072	0	1
Discr. Sexuality	52,458	0.003	0.050	0	1
Discr. Disability	52,458	0.005	0.071	0	1
Discr. Other	52,458	0.014	0.117	0	1
Gender	52,437	1.546	0.498	1	2
Age	52,305	48.505	18.789	14	102
Marital status	50,059	2.155	1.310	1	4
Habitat size	52,343	2.235	1.171	1	4
Education level	52,198	2.767	0.919	1	4
Household income	39,838	2.314	1.175	1	4

Once the statistical relevance at the bivariate level had been determined between the predictors and the dependent variable, the multilevel ordinal regression model was carried out.

### Specification of the multilevel ordered logit model

Given that our response variable, self-rated health, is an ordinal with 5 possible values and the hierarchical structure of individuals (level-1) clustered within country units (level-2), a multilevel ordered logit model was carried out to work with this nested structure [29]. This specific analysis enables variations in self-rated health related to an individual and his/her context to be explored simultaneously. It should be noticed that, on the one hand, in the present study only random intercept effects are considered, so correlations between any pair of repeated measures are equal. On the other, level 2 has no explanatory variables since our study is specifically aimed to describe variations between countries but not to describe interactions between individual and contextual levels.

The multilevel ordered logit model is the following:

logit{Pr(y_ij_≤m}=τ_m_−x'_ij_β+u_j_,

where *y*_*ij*_ is the observed ordinal response of the *i*_*th*_ individual unit (i.e. subjects interviewed) nested within the *j*_*th*_ cluster (i.e. countries) for the corresponding explanatory variables *x*_*ij*_ in the model, and *u*_*j*_ represents the random intercept effect normally distributed with a mean zero and variance . In this model, the effect of every *x*_*ij*_ independent variable is assumed to be fixed across country units, but there is a random intercept that considers the variations in responses between the countries in the survey (2010). Then errors are considered to be constant and are not correlated between country units. Restricted Maximum Likelihood is used to estimate the multilevel ordered logit model.

Finally, Brant’s test [30] for proportional odds was assessed to provide evidence that the parallel regression assumption had not been violated. This test indicates that our statistical model can be adequately performed.

## Results

Tables 2-3 show the prevalence of perceived discrimination in 27 EU countries (ESS, 2010). Great Britain appears as the country with the highest percentage of perceived discrimination in Europe (11.6%), while –excluding Israel as an EU country- Cyprus presents the lowest (2.6%). Females perceive more discrimination than males in Eastern countries such as Slovak Republic (71.6%), Lithuania (69.0%), Ukraine (66.2%), and Estonia (63.9%). However, the perceived discrimination of females is relevant in Mediterranean and Northern European countries such as Greece (61.1%) or Finland (57.0%). Age discrimination seems to be higher between Eastern/Post-communist countries, especially between people over 55 years old. Perceived discrimination seems to be higher among people living in big cities of Eastern/post-communist countries such as Lithuania (61.4%) or Estonia (50.9%), and also in Mediterranean countries such as Portugal (51.3%) and Greece (42.7%). Meanwhile in Northern European countries more discrimination is perceived among individuals living in small cities, and generally among people living together, both married and in civil union. Finally, in relation to individual socio-economic status, it is seen that people with low income and (upper) secondary education perceive more discrimination. The above variations in countries’ percentages are statistically significant at the level of p<0.01, which confirms our first hypothesis (H1).

**Table 2:** Prevalence of perceived discrimination in Europe (ESS, 2010) (in percentages).

**Variable**		**Belgium**	**Bulgaria**	**Switzer-land**	**Cyprus**	**Czech Republic**	**Germany**	**Denmark**	**Estonia**	**Spain**	**Finland**	**France**	**Great Britain**	**Greece**	**Croatia**
**Discriminated?**	**Yes**	4.82	9.71	4.39	2.59	6.20	4.24	4.33	6.14	4.53	8.80	9.41	11.57	7.50	6.69
**Gender**	**Male**	54.88	45.73	62.12	50.00	46.26	50.78	52.94	36.11	54.12	43.03	46.91	46.95	38.92	55.24
	**Female**	45.12	54.27	37.88	50.00	53.74	49.22	47.06	63.89	45.88	56.97	53.09	53.05	61.08	44.76
**Age interval**	**15-24**	12.20	7.26	15.15	21.43	4.76	21.09	14.71	12.96	15.29	12.12	12.35	11.19	12.32	6.73
	**25-34**	17.07	16.67	16.67	17.86	13.61	23.44	11.76	10.19	25.88	20.61	19.14	15.52	21.18	7.69
	**35-44**	20.73	17.95	28.79	21.43	16.33	17.97	23.53	19.44	21.18	13.33	27.16	21.66	22.66	19.23
	**45-54**	20.73	14.53	13.64	21.43	20.41	23.44	23.53	18.52	22.35	18.18	23.46	16.97	17.24	12.50
	**55-64**	13.41	22.65	18.18	7.14	23.13	6.25	19.12	15.74	5.88	20.61	10.49	18.77	8.37	26.92
	**65-**	15.85	20.94	7.58	10.71	21.77	7.81	7.35	23.15	9.41	15.15	7.41	15.88	18.23	26.92
**Education level**	**Primary**	13.58	25.21	7.58	14.29	0.68	5.56	11.76	2.78	16.47	15.76	15.53	18.82	36.45	11.43
	**Lower secondary**	28.40	30.34	21.21	10.71	17.01	20.63	23.53	14.81	24.71	14.55	11.18	13.65	12.81	14.29
	**Upper secondary**	28.40	30.34	56.06	57.14	74.15	57.94	30.88	56.48	34.12	50.30	50.93	44.28	37.44	58.10
	**Tertiary**	29.63	14.10	15.15	17.86	8.16	15.87	33.82	25.93	24.71	19.39	22.36	23.25	13.30	16.19
**Household income**	**Q1**	40.00	72.00	31.03	61.11	36.94	45.79	23.33	43.68	26.47	47.37	46.26	41.63	56.74	29.87
	**Q2**	17.14	12.50	18.97	11.11	27.93	19.63	16.67	19.54	26.47	15.79	21.77	14.48	28.37	23.38
	**Q3**	22.86	7.50	24.14	11.11	24.32	18.69	31.67	19.54	13.24	15.79	17.01	19.91	12.06	16.88
	**Q4**	20.00	8.00	25.86	16.67	10.81	15.89	28.33	17.24	33.82	21.05	14.97	23.98	2.84	29.87
**Domicile**	**Rural village**	40.24	35.47	36.36	21.43	26.53	26.56	16.18	10.19	34.12	37.20	26.54	19.78	27.59	28.30
	**Small city**	23.17	28.63	39.39	32.14	31.97	38.28	26.47	32.41	25.88	32.93	32.72	45.68	17.73	27.36
	**Suburbs big city**	14.63	3.42	9.09	10.71	7.48	10.94	30.88	6.48	9.41	12.80	12.96	25.18	11.82	13.21
	**Big city**	21.95	32.48	15.15	35.71	34.01	24.22	26.47	50.93	30.59	17.07	27.78	9.35	42.86	31.13
**Marital status**	**Married/civil union**	48.78	44.02	59.09	42.31	51.03	45.67	51.47	48.15	52.94	-	38.27	42.45	51.72	65.00
	**Divorced**	13.41	10.68	10.61	11.54	12.41	14.96	10.29	12.96	8.24	-	16.67	18.71	6.90	5.00
	**Widowed**	6.10	18.80	1.52	7.69	15.86	1.57	1.47	13.89	0.00	-	4.94	7.19	10.84	12.00
	**Single**	31.71	26.50	28.79	38.46	20.69	37.80	36.76	25.00	38.82	-	40.12	31.65	30.54	18.00
**Sample Size**	**N**	1,620	2,176	1,436	1,052	2,223	2,889	1,502	1,650	1,792	1,711	1,560	2,133	2,502	1,507

Note: All variables are statistically significant at p<0.01.

**Table 3:** Prevalence of perceived discrimination in Europe (ESS, 2010) (in percentages).

**Variable**		**Hungary**	**Ireland**	**Israel**	**Lithuania**	**Netherl-and**	**Norway**	**Poland**	**Portugal**	**Russia**	**Sweden**	**Slovenia**	**Slovak Republic**	**Ukraine**
**Discriminated?**	**Yes**	6.37	5.10	16.48	3.53	7.96	5.37	4.41	3.60	8.00	7.24	3.02	3.66	4.13
**Gender**	**Male**	47.47	57.25	58.31	31.03	44.14	50.60	59.74	44.16	44.22	35.19	52.38	28.36	33.77
	**Female**	52.53	42.75	41.69	68.97	55.86	49.40	40.26	55.84	55.78	64.81	47.62	71.64	66.23
**Age interval**	**15-24**	18.18	21.37	17.86	8.77	13.79	14.46	16.88	12.99	9.55	17.59	19.51	11.94	14.29
	**25-34**	21.21	29.77	30.77	8.77	14.48	16.87	18.18	19.48	17.59	17.59	19.51	19.40	10.39
	**35-44**	22.22	24.43	17.31	19.30	20.00	18.07	16.88	23.38	15.58	16.67	14.63	10.45	11.69
	**45-54**	17.17	14.50	16.21	19.30	21.38	21.69	19.48	18.18	19.10	11.11	26.83	26.87	14.29
	**55-64**	11.11	5.34	10.44	21.05	19.31	16.87	23.38	10.39	17.59	20.37	12.20	16.42	22.08
	**65-**	10.10	4.58	7.42	22.81	11.03	12.05	5.19	15.58	20.60	16.67	7.32	14.93	27.27
**Education level**	**Primary**	9.09	14.52	6.83	5.26	10.42	1.20	2.60	38.96	5.53	9.26	10.00	4.48	3.90
	**Lower secondary**	36.36	23.39	11.75	17.54	37.50	16.87	42.86	20.78	8.54	13.89	22.50	14.93	11.69
	**Upper secondary**	46.46	37.10	52.46	45.61	32.64	45.78	27.27	29.87	59.30	50.93	47.50	61.19	53.25
	**Tertiary**	8.08	25.00	28.96	31.58	19.44	36.14	27.27	10.39	26.63	25.93	20.00	19.40	31.17
**Household income**	**Q1**	51.22	75.58	27.20	51.11	43.22	35.44	40.00	-	43.71	39.18	33.33	37.50	54.41
	**Q2**	20.73	11.63	35.63	24.44	24.58	26.58	18.46	-	22.16	16.49	23.08	18.75	14.71
	**Q3**	12.20	10.47	22.22	11.11	17.80	13.92	10.77	-	16.77	11.34	23.08	20.83	19.12
	**Q4**	15.85	2.33	14.94	13.33	14.41	24.05	30.77	-	17.37	32.99	20.51	22.92	11.76
**Domicile**	**Rural village**	42.42	22.14	19.89	10.53	30.34	48.19	27.27	7.89	23.12	28.97	31.71	36.36	28.57
	**Small city**	26.26	36.64	34.88	24.56	29.66	21.69	33.77	21.05	37.69	31.78	36.59	33.33	32.47
	**Suburbs big city**	8.08	28.24	7.63	3.51	13.79	13.25	3.90	19.74	5.03	21.50	19.51	4.55	1.30
	**Big city**	23.23	12.98	37.60	61.40	26.21	16.87	35.06	51.32	34.17	17.76	12.20	25.76	37.66
**Marital status**	**Married/civil union**	40.40	35.43	63.91	39.29	41.84	44.58	50.67	46.75	38.38	36.11	31.58	49.25	45.33
	**Divorced**	17.17	12.60	3.86	21.43	18.44	13.25	4.00	9.09	19.19	18.52	13.16	10.45	21.33
	**Widowed**	5.05	2.36	4.13	16.07	3.55	2.41	5.33	10.39	19.70	4.63	5.26	8.96	17.33
	**Single**	37.37	49.61	28.10	23.21	36.17	39.76	40.00	33.77	22.73	40.74	50.00	31.34	16.00
**Sample Size**	**N**	1,454	2,436	1,860	1,587	1,677	1,463	1,668	2,062	2,290	1,384	1,348	1,765	1,789

Note: All variables are statistically significant at p<0.01.

These initial findings suggest the existence of differences in Europeans’ general perceived discrimination. But, according to our second hypothesis, the question is: how are different types of perceived discrimination related to different health outcomes in European countries? That is, how can different forms of perceived discrimination affect Europeans’ self-rated health?

The multilevel ordered logit model was performed using Stata’s GLLAMM (Generalized Linear and Latent Mixed Models). In this model we assume that underlying there exist a latent variable that captures individual predisposition to report specific values in SRH. Thus, if the latent response exceeds a certain threshold (i.e. cutpoint), then the individual picks a particular value in the dependent variable. In addition, the latent variable is defined by a two-level hierarchical structure: subjects *i* (level-1) nested in countries *j* (level-2). In the study only random intercept effects are considered (i.e. variation between countries). In other words, we are assuming that there is a contextual effect that produces differences in the association between different types of perceived discrimination and SRH for specific countries. The estimated random-intercept variance is initially low, giving an estimated intraclass correlation coefficient of 0.10 (= 0.38/(0.38 + π^2^/3)). This result indicates that SRH of European countries is relatively similar; however, there is a 10% of the overall variation of our dependent variable that is related with contextual level (i.e. country differences).

Table 4 describes three multilevel ordered logistic models that aim to compare the effect of different types of perceived discrimination on self-rated health: Model 1 unadjusted; Model 2, adjusted by socio-demographic variables; Model 3, adjusted by socio-demographic and socio-economic variables. In these models, results are presented as proportional odds ratios (OR) (i.e. exponentiated coefficients). The goodness-of-fit test was appropriate and statistically significant for these models. Variables in the equation did not present multicollinearity and have no effect on the overall test of the model or on model predictions.

**Table 4:** Perceived discrimination in Europe. Multilevel Ordered Logit.

	**Model 1.**	**Model 2.**	**Model 3.**
	**Unadjusted model**	**Adjusted by socio-demographic var.**	**Adjusted by socio-economic var.**
***Perceiveddiscrimination***			
Color or race	1.16 (0.10)	0.84 (0.08)^*^	0.93 (0.10)
Nationality	1.20 (0.10)^*^	0.89 (0.08)	0.95 (0.10)
Religion	1.20 (0.11)^*^	1.05 (0.10)	0.96 (0.11)
Language	0.94 (0.11)	1.10 (0.14)	1.01 (0.15)
Ethnic group	1.02 (0.09)	0.79 (0.08)^**^	1.02 (0.11)
Age	0.37 (0.032)^***^	0.63 (0.06)^***^	0.67 (0.07)^***^
Gender	1.31 (0.15)^*^	1.00 (0.12)	0.94 (0.13)
Sexuality	1.01 (0.16)	0.61 (0.11)^**^	0.60 (0.12)^**^
Disability	0.10 (0.01)^***^	0.07 (0.10)^***^	0.08 (0.01)^***^
Other	0.70 (0.05)^***^	0.57 (0.04)^***^	0.55 (0.05)^***^
***Gender(malereferencecat.)***			
Female		0.84 (0.02)^***^	0.87 (0.02)^***^
***Age(15–24)***			
Age 25-34		0.66 (0.02)^***^	0.64 (0.03)^***^
Age 35-44		0.39 (0.02)^***^	0.38 (0.02)^***^
Age 45-54		0.21 (0.01)^***^	0.21 (0.01)^***^
Age 55-64		0.13 (0.01)^***^	0.15 (0.01)^***^
Age 65-		0.07 (0.01)^***^	0.09 (0.01)^***^
***Maritalstatus(marr./c.union)***			
Divorced		0.78 (0.02)^***^	0.92 (0.03)^**^
Widowed		0.59 (0.02)^***^	0.79 (0.03)^***^
Single		0.86 (0.02)^***^	0.96 (0.03)
***Domicile(ruralvillage)***			
Domicile: Small city		1.01 (0.02)	0.95 (0.02)^*^
Domicile: Suburbs big city		1.14 (0.03)^***^	1.03 (0.04)
Domicile: Big city		1.12 (0.03)^***^	0.94 (0.03)^*^
***Education(primary)***			
Lower secondary			1.40 (0.06)^***^
Upper secondary			1.85 (0.07)^***^
Tertiary			2.52 (0.11)^***^
***Householdincome(Q1)***			
Income Q2			1.42 (0.04)^***^
Income Q3			1.61 (0.05)^***^
Income Q4			2.03 (0.06)^***^
**Threshold 1**	-4.22 (0.12)^***^	-6.37 (0.14)^***^	-5.39 (0.16)^***^
**Threshold 2**	-2.38 (0.12)^***^	-4.41 (0.14)^***^	-3.43 (0.16)^***^
**Threshold 3**	-0.57 (0.12)^***^	-2.29 (0.14)^***^	-1.25 (0.16)^***^
**Threshold 4**	1.29 (0.12)^***^	-.084 (0.14)^***^	0.99 (0.16)^***^
**Log-likelihood**	-67,237.80	-57,837.14	-43,327.60
**Variance Level-2 (U_0j_)**	0.38 (0.10)	0.46 (0.13)	0.53 (0.15)
**No Observ. Level-1**	52,379	49,754	37,476
**No Observ. Level-2**	27	26	25

Note: exp (b) and std. error in brackets. Significance levels: ^***^ p<0.001, ^**^p<0.01, ^*^p<0.05.

Note2: the sample size is reduced by the income variable.

Model 1 confirms the suggestion regarding the effect of different types of perceived discrimination on self-rated health of people living in Europe. In other words, different forms of discrimination are associated to different health outcomes in European countries (H2). Thus the relationship between the individual and the contextual level is confirmed. In this model, only perceived discrimination due to ‘nationality’, ‘religion’, ‘age’, ‘gender’, ‘disability’ and ‘other’ are statistically significant. For instance, perceived discrimination due to age (OR = 0.37) and disabilities (OR =0.10) reduce the probabilities of reporting a good state of health, like when using other (OR = 0.70) types of discrimination that have not been specifically reported in this survey (and possibly related to socio-economic background: income level, education, occupational status, etc.). On the other hand, gender, nationality and religious discrimination increase the probabilities of reporting a positive health outcome.

Model 2 adds socio-demographic variables to control the effect of perceived discrimination (gender, age, marital status and domicile size). The introduction of these predictors enables the initial OR to be better defined. In this case, perceived discrimination by ‘color/race’ (OR = 0.84) and ‘ethnic’ (OR = 0.79) become statistically significant, while ‘gender’ discrimination does not present differences between countries. Perceived discrimination due to age (OR = 0.63), disability (OR = 0.07) and other (OR = 0.57) remain statistically significant with respect to the previous model. That is, these types of perceived discrimination reduce the probability of reporting a positive health outcome in European countries. These findings confirm our third hypothesis regarding the relevance of age and disability discrimination in Europe (H3). Additionally, in this model adjusted by socio-demographic variables, ‘sexual’ discrimination generates statistically significant differences between countries (OR = 0.61). In this case, this type of perceived discrimination reduces the probability of reporting a good state of health.

In reference to control variables, compared to males, females are found to report a worse state of health more frequently, as do older people. On the other hand, people living together (i.e. married or in civil union) or residing in big cities are more likely to report positive health outcomes.

Model 3 is adjusted both by socio-demographic and socio-economic variables such as education and income. These two variables have been included in a second step in order to understand how socio-economic status may vary the association between perceived discrimination and SRH. In this model, the OR remain relatively constant with respect to the above, with the exception of the effect of perceived discrimination by ‘ethnic group’ that loses its statistical significance. In this model, perceived discrimination due to ‘age’ (OR = 0.67), ‘sexuality’ (OR = 0.60), ‘disability’ (OR = 0.08) and ‘others’ (OR = 0.55) are the factors that generate the most health-related inequalities in European countries. This means that geographically, we can locate differences in the effect of these health determinants on self-rated health in European countries. Furthermore, among these different types of perceived treatment inequity, ‘disability’ discrimination is the one that most reduces the probability of reporting a good state of health.

As could be expected from previous studies, socio-economic status has a positive effect on self-rated health. That is, positive assessment of health is more frequent among individuals with a higher level of education or household income.

Now the question is the following: which are the countries where ‘age’, ‘disability’ and ‘sexual’ discrimination present a higher effect on self-rated health? To answer this question, Model 3 has been used to calculate the combined probabilities of perceiving discrimination due to these factors and yet reporting of a positive health outcome, that is, a good state of health (i.e. values of 4 ‘good’ or 5 ‘very good’ on the scale of self-rated health).

**Figure 1:**
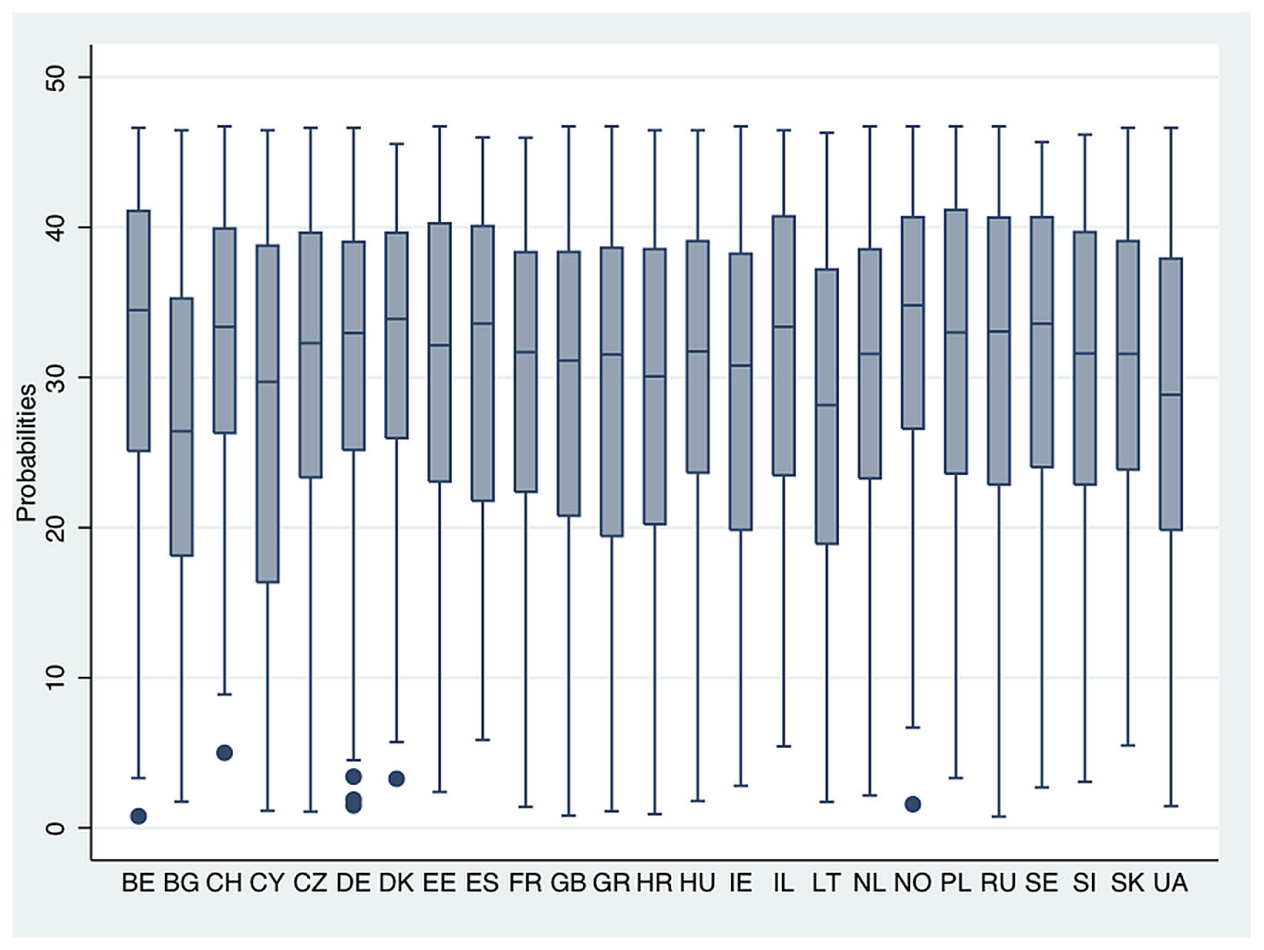
Predicted probabilities of people being discriminated by age, disability or sexuality report good health in Europe.

Figure 1 shows predicted probabilities of people being discriminated by age, disability or sexuality report good health in Europe. Now it is seen how the probabilities that discriminated people report a positive state of health reach about 30%, despite not finding big differences among European countries. Obviously, it must be taken into account that groups that perceive themselves as being discriminated against represent a minority among European populations (and even more so if we look at specific types of discrimination), so huge differences cannot be expected. Of course, it is possible to observe how Eastern/Post-communist countries such as Ukraine, Lithuania or Bulgaria are those where these probabilities are lower while, on the other hand, Northern and Central European countries (e.g. Norway, Sweden, Denmark, Belgium or Switzerland) are more likely to report a positive health outcome. In other words, the effect of perceived discrimination over SRH seems to be lower between countries of developed welfare state systems, which are characterized by comparatively generous social transfers and a wide redistributive social security system.

Additionally, it is possible to find some peculiar countries such as Cyprus or Spain. For instance, in Cyprus, the effect of perceived discrimination over SRH seems to be similar than in European Eastern countries (i.e. present a higher effect over poor health outcomes), while the effect in Spain is similar to Scandinavian countries (i.e. a lower effect).

## Discussion

This study compares the relationship between different experiences of discrimination and self-rated health in European countries. The present work describes the prevalence of perceived discrimination in Europe, and identifies the main causes of perceived discrimination that can be associated with health-related inequalities.

In agreement with previous studies, our work confirms that the prevalence of perceived discrimination in Europe varies depending on socio-demographic and socio-economic determinants like gender, age, marital status, education, household income or domicile size. Although both types of factors generate statistically significant differences on perceived discrimination, it is interesting to note the relevance of socio-economic determinants such as income and education on reducing the perception of negative treatment towards certain socially excluded groups, and ultimately to decrease health-related inequalities [31].

Our findings suggest that different forms of perceived discrimination are related to different health outcomes in Europe. That is, the multilevel ordered model we have performed identify statistically significant differences in the effect that diverse types of perceived discrimination can generate on the self-rated health of Europeans. Specifically, our study identifies three well-defined types of perceived discrimination that can be associated with poor health outcomes: (1) age discrimination; (2) disability discrimination; and (3) sexuality discrimination. As hypothesized, the effect of perceived discrimination related to aging and disabilities on self-rated health seems to be more relevant than other types of discrimination in the European context with a longer tradition in the literature (e.g. ethnic and/or race-based). These results reinforce the argument about the persistence of aging and disability problems that need to be solved in advanced post-industrialized societies [25–27], and especially when these can affect both the mental and physical integrity of European citizens.

Obviously, before the designing of appropriate social policies to avoid these problems, it is needed to study what are the factors related to these concrete forms of discrimination and how these forms may be associated with poor health outcomes. For instance, behind the problem of age discrimination we might identify differences between ‘middle age’ individuals that feel discriminated against when job hunting, and the retired elderly that may perceive other types of discrimination. In this sense, age discrimination is found not only in the labor market but also in other areas of everyday life, for example, those related to access to welfare-state benefits such as pensions or health services [32]. On the other hand, there is a need to research the multiple forms of discrimination against people with mental and physical disabilities, and thus the causes that allow them to persist in so-called ‘advanced’ societies [24]. In short, it is evident that, compared to other types of inequity based on gender or ethnical stereotypes, both age and disability discrimination have become an invisible part of our everyday lives.

In addition, according to the findings of previous studies [33–35], results in our model have highlighted the relevance of sexual discrimination as being related to poor health outcomes in Europe. Like age or disability forms of intolerance, sexuality-based discrimination represents a clear determinant of mental and physical health in current societies since these groups experience different medical care access and treatment.

Of course, taking into account the variability of these types of perceived discrimination different political actions should be carried out to eradicate these complex problems. Age discrimination is partially related with welfare state policies and labor market protection, therefore European governments should concentrate on problems such as the employability of middle-aged and unskilled workers and, for instance, the maintenance of the pensions system for elderly and people with physical or intellectual disabilities. Thus to eradicate health inequities, and inequitable conditions related with discrimination, it is necessary a strong public sector that provides equity in resources for these disadvantaged groups and the support and tolerance of civil society to address the persistent problems of discrimination and social exclusion [36]. Obviously, we cannot guarantee the eradication of discrimination and social exclusion in our societies, however, educational policies can play an important role in reducing difficulties for social inclusion and health inequalities [31,36].

As observed, the effect of perceived discrimination over SRH seems to be lower between countries of developed welfare state systems (Nordic/Social-democratic, Anglo-Saxon/Liberal and Central European/Conservative) [37,38]. Especially, in Nordic countries, which are characterized by comparatively generous social transfers and a wide redistributive social security system, the impact of perceived discrimination is clearly reduced and health inequalities are lower. A similar trend is followed by Liberal (e.g. United Kingdom) and Conservative countries (e.g. Germany or France). While, on the contrary, the relationship of discrimination with health inequalities is higher between Mediterranean (excluding Spain) and Eastern countries, those characterized by a fragmented and underdeveloped system of public provision [38]. Among these countries Spain represents the exception and this is possibly due to the effect of the public and universal health system in this country. Despite this association should be specifically analyzed, this could be an argument about the need of public intervention over health inequalities. Of course, discrimination should also be addressed with specific educative policies; however, health care should be a universal guaranty, especially for excluded groups that generally compete in unequal conditions (e.g. in labor markets) [37].

This study faces three basic limitations that should be highlighted and addressed in future studies. First, this study cannot define the causation between perceived discrimination and self-rated health since a cross-sectional sample of ESS 2010 data is being used. In this work we have described and compared the association between these variables, however, we cannot establish the linear causation among them. Literature has focused on perceived discrimination as a determinant of health outcomes, but this assumption can be inverted. Then the initial question would be reformulated: “Can different health outcomes affect perceived discrimination?” or, for example, “how can different health-related inequalities affect individuals’ perceived discrimination?”

Second, in future studies, separate models would have to be created to specifically analyze what is behind these different types of discrimination and the specific situations of different social groups. The elderly, people with disabilities, homosexuals, immigrants or working/lower classes are different social groups characterized by specific interest. Subsequently, specific studies should address the concerns of these groups and how these worries may generate a poor health outcome or, conversely, analyze how a poor state of health can modify the specific interests of these groups. Of course, to do that, we need to perform specific surveys that provide specific information about these different groups.

Finally, future works should include other types of perceived discrimination due to socio-economic factors such as education, income or occupational status. Our analysis includes perceived discrimination by ‘others’ as an umbrella category. Although this factor appears in the analysis as being statistically significant, we cannot infer what is behind this general response.

## Conclusions

This work shows that the prevalence of perceived discrimination in Europe varies depending on socio-demographic and socio-economic determinants. Particularly as the main result, it also indicates that experiences of discrimination based on factors such as age, disabilities and sexuality are related to poor health outcomes in Europe. In other words, differences in health equity among European population are associated to variations in these types of perceived discrimination.

The WHO Commission on Social Determinants of Health (CSDH) have recently defined the concept of health equity as “the absence of unfair and avoidable or remediable differences in health among population groups defined socially, economically, demographically or geographically” [36]. In this line, our study has followed this orientation in order to identify and compare health inequalities in Europe. This work gives echo to the existence of health inequities that can still be found throughout European countries and between different groups of people living therein. On the other hand, the present study shows that the relationship between perceived discrimination and health inequities in Europe is not random, but systematically distributed depending on factors such as age, sexuality and disabilities. Therefore the future orientation of EU social policies should aim to reduce the impact of these social determinants on health equity, and especially to protect discriminated social groups against the current context of financial crisis where socio-economic inequalities and scarcity are more likely to produce poor health outcomes.
